# Reliability of Modified Radiographic Union Score for Tibia Scores in the Evaluation of Femoral Shaft Fractures in a Low-resource Setting

**DOI:** 10.5435/JAAOSGlobal-D-21-00211

**Published:** 2022-05-20

**Authors:** Mayur Urva, Sravya T. Challa, Billy T. Haonga, Edmund Eliezer, Zachary M. Working, Ashraf El Naga, Saam Morshed, David W. Shearer

**Affiliations:** From the Institute for Global Orthopaedics and Traumatology, University of California, San Francisco San Francisco, CA (Mr. Urva, Dr. Morshed, and Dr. Shearer); Harvard Combined Orthopaedic Residency Program, Boston, MA (Dr. Challa); Muhimbili Orthopaedic Institute, Dar es Salaam, Tanzania (Dr. Haonga and Dr. Eliezer); Oregon Health & Sciences University, Portland, OR (Dr. Working); and University of California, San Francisco, San Francisco, CA (Dr. El Naga).

## Abstract

The modified Radiographic Union Score for Tibia (RUST) fractures was developed to better describe fracture healing, but its utility in resource-limited settings is poorly understood. This study aimed to determine the validity of mRUST scores in evaluating fracture healing in diaphyseal femur fractures treated operatively at a single tertiary referral hospital in Tanzania.

**Methods:**

Radiographs of 297 fractures were evaluated using the mRUST score and compared with outcomes including revision surgery and EuroQol five dimensions questionnaire (EQ-5D) and visual analog scale (VAS) quality-of-life measures. Convergent validity was assessed by correlating mRUST scores with EQ-5D and VAS scores. Divergent validity was assessed by comparing mRUST scores in patients based on revision surgery status.

**Results:**

The mRUST score had moderate correlation (Spearman correlation coefficient 0.40) with EQ-5D scores and weak correlation (Spearman correlation coefficient 0.320) with VAS scores. Compared with patients who required revision surgery, patients who did not require revision surgery had higher RUST scores at all time points, with statistically significant differences at 3 months (2.02, *P* < 0.05).

**Discussion:**

These results demonstrate that the mRUST score is a valid method of evaluating the healing of femoral shaft fractures in resource-limited settings, with high interrater reliability, correlation with widely used quality of life measures (EQ-5D and VAS), and expected divergence in the setting of complications requiring revision surgery.

The global burden of trauma is increasing rapidly,^1^ particularly in low- and middle-income countries (LMICs), where long-bone fractures and other musculoskeletal injuries cause notable disability and mortality.^2–4^ Owing to resource limitations in these settings, long-bone fractures can result in long-term sequelae that are often preventable in high-resource settings, where the established treatment guidelines are more locally applicable. Improving research capacity in LMICs is essential to establishing the contextually appropriate guidelines of care for these injuries.^2,5^

One aspect of musculoskeletal research in which LMICs face unique challenges is outcome assessment, particularly radiographic evaluation. Hospitals in LMICs have less access to basic medical imaging, and the radiologic resources that do exist are often less modern than those in high-income countries.^6^ With photographs of plain radiographs, there is no ability to adjust contrast or to “zoom in” on the fracture site, making evaluation of bony healing more challenging compared with settings with access to digital picture archiving and communication system.

Although there exists no universally accepted measure of bony healing, radiographic union is a commonly referenced end point of successful fracture treatment.^7^ The definition of union remains poorly defined, but recent image-based assessments such as the Radiographic Union Score for Tibia (RUST) score and modified RUST (mRUST) have shown promise in better representing the spectrum of fracture healing.^7,8^ These scoring systems have proven to be useful measures of physical and biomechanical healing in tibial fractures,^9–11^ but few studies have assessed their applicability to fractures at other sites and in low-resource settings. Given that the score addresses callus development in healing fractures, this system should be generalizable to most other long bones. A recent study found the modified RUST scoring system to be reliable in assessing radiographic union in metadiaphyseal femoral fractures in North America.^8,12^ However, few studies have compared the mRUST score in femoral shaft fractures with existing patient-reported outcome measures.

We sought to determine whether the interrater reliability of the mRUST scoring of femoral fractures would be sufficiently high to warrant its use in clinical research at a tertiary care center in Tanzania, where the scores were obtained using uploaded photographs of printed plain radiographs on a light box. We also sought to assess the correlation of the mRUST scores with validated quality of life instruments, including the EuroQol five dimensions questionnaire (EQ-5D) and visual analog scale (VAS) analog scale, as well as the correlation of the mRUST scores with the development of complications ultimately requiring revision surgery. As an additional exploratory outcome, in cases where only a single-view radiograph was obtained due to resource constraints, we sought to compare the single-view, two-cortex score with patient-reported quality of life and revision surgery status. We hypothesize that the mRUST scores will exhibit high interrater reliability and will correlate with both patient-reported quality-of-life measures and with complications requiring revision surgery.

## Methods

### Study Population and Design

**Figure 1:**
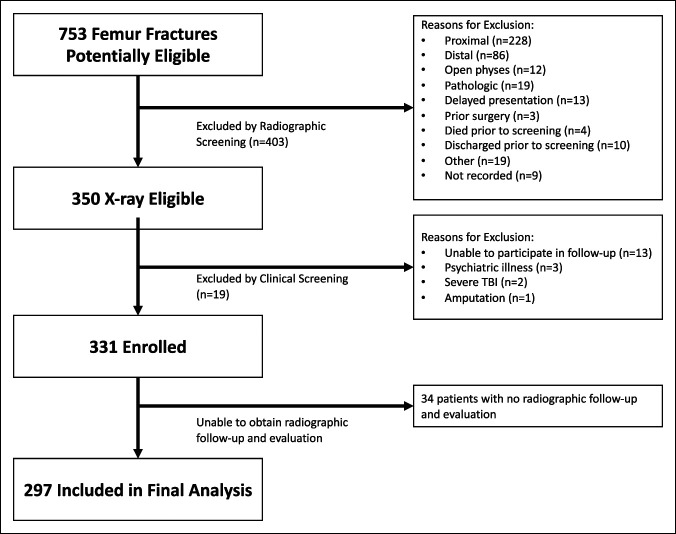
Flowchart demonstrating screening, enrollment, and follow-up for study participants. TBI = traumatic brain injury.

This was an unplanned secondary analysis of a previously published prospective observational study.^8^ The original study enrolled 329 patients with diaphyseal femur fractures (331 fractures) treated operatively at a tertiary referral hospital in Dar es Salaam, Tanzania, from July 2012 to July 2013. The exclusion criteria for the original study population were (1) skeletal immaturity, (2) pathologic fracture, (3) previous surgery involving the affected femur, (4) delayed presentation (≥6 weeks from injury), (5) active infection at the surgical site, (6) severe traumatic brain injury, (7) severe burns, and (8) the inability to participate in the follow-up. Patients were treated at surgeon discretion using intramedullary nailing, plate fixation, or external fixation. This study included the 297 patients (of the original 329) who had adequate radiographic follow-up and evaluation (Figure 1). The study population consisted primarily of young, healthy men who sustained isolated femoral shaft fractures from road traffic injuries (Table 1). We considered a patient to have an adequate follow-up if postoperative radiographs were obtained at a minimum of one follow-up visit (at 6 weeks, 3 months, or 6 months), and the uploaded images were of sufficient quality to assess cortical bridging.

**Table 1:** Patient Characteristics^a^

Factor	Total (N = 293)
Age^b^	32.02 ± 11.2
Sex, n (%)	
Male	251 (85.7)
Female	42 (14.3)
Formal employment, n (%)	
Yes	86 (29.5)
No	206 (70.5)
Mechanism of injury, n (%)	
Motor vehicle crash	139 (49.5)
Motorcycle crash	99 (35.2)
Pedestrian struck by automobile	6 (2.1)
Fall	31 (11)
Crush	6 (2.1)
Injury Severity Score^b^	9.06 ± 0.3
Any comorbidity, n (%)	7 (2.4)
Smoker, n (%)	
Current	39 (13.4)
Former	33 (11.3)
Never	219 (75.3)
Alcohol use, n (%)	
No	203 (70)
Yes	87 (30)
Side, n (%)	
Left	114 (39.2)
Right	172 (59.1)
Bilateral	5 (1.7)
BMI, n (%)	
<18 kg/m^2^	8 (2.8)
18-25 kg/m^2^	209 (7.2)
>25-30 kg/m^2^	54 (18.7)
>30 kg/m^2^	18 (6.2)
Baseline EQ-5D index^b^	0.996 ± 0.04
Baseline EQ-5D VAS^b^	99.2 ± 3.3
Time to presentation^c^ (d)	1 (0-1)
Time to surgery^c^ (d)	7 (3-13)

BMI = body mass index, EQ-5D = EuroQol five dimensions questionnaire, VAS = visual analog scale

a A total of 297 participants included in this study. Some demographic data were not reported for some patients.

b The values are given as the mean ± SD.

c The values are given as the median, with the interquartile range in parentheses.

Plain radiographs were taken preoperatively and postoperatively and at follow-up appointments at 6 weeks, 3 months, and 6 months after surgery, and photographs of the plain radiographs were uploaded to REDCap electronic data capture tools hosted at the partner US Institution.^13^ Patients were followed clinically for revision surgery and complications for up to 1 year after surgery. In total, there were 192 patients who had at least one radiograph image that was scored by the reviewers at 6 weeks, 167 at 3 months, and 112 at 6 months. Both AP and lateral radiograph images of 80 patients were scored at 6 weeks, 86 at 3 months, and 59 at 6 months. The EQ-5D and VAS were administered at all time points, and all complications were recorded and adjudicated by the committee. All cases in which revision surgery was recommended by the adjudication committee were considered a study event, regardless of whether the revision surgery actually occurred.

### Modified and Single-view Radiographic Union Score for Tibia Scoring

Two fellowship-trained orthopaedic trauma surgeons (A.N.E., Z.M.W.) who were blinded to the study outcomes assessed the uploaded pictures of the radiographs and determined the modified RUST score according to the parameters described by Litrenta et al,^12^ where each cortex is scored from 1 to 4 (1 = no callus, 2 = callus present, 3 = bridging callus, and 4 = remodeled with no visible fracture) for a total score ranging from 4 to 16. In addition, given the challenges of obtaining orthogonal images of the fracture in all cases because of resource limitations, an abbreviated, single-view RUST scoring system was developed. For patients with only one radiograph view, the score of that individual radiograph was recorded as a single-view RUST score (anterior/posterior or medial/lateral, depending on which cortices were visible in the radiograph). In patients with both radiograph views obtained, both the single-view scores (ranging from 2 to 8) and the modified RUST score (ranging from 4 to 16) were recorded.

### Validity of Radiographic Union Score for Tibia Scores for Femoral Shaft Fractures

Statistical analysis was conducted using Stata (version 16.0, StataCorp). Convergent and divergent validities were assessed. Convergent validity refers to the extent to which two similar constructs correspond to one another. Spearman correlation coefficients (SCCs) were determined by comparing RUST scores with both EQ-5D indices and VAS scores at each time point; convergent validity was considered to be present if the SCC demonstrated at least fair correlation (SCC ≥0.30).^14^ Divergent validity is the ability of a particular measurement tool to distinguish between different groups or constructs within its own data. A two-sample *t*-test was used to compare the means of the RUST at all relevant follow-up visits (6 weeks, 3 months, 6 months, and 1 year) between two groups: patients requiring revision surgery and patients not requiring revision surgery. The Levene test for equality of variances was used to determine whether the homogeneity of variance could be assumed between the two groups, and the appropriate *t*-test assuming equal or unequal variance was done accordingly. Divergent validity was considered to be present if the mean scores between patients requiring revision surgery and those not requiring revision surgery were statistically different with *P* < 0.05. Kappa statistic was used to calculate interrater reliability of the RUST and single-view RUST scores.

### Correlating Single-view Radiographic Union Score for Tibia Scores With Modified Radiographic Union Score for Tibia Score

To determine the validity of the single-view RUST scores, individual view scores were correlated with modified RUST scores for 268 patient encounters where both radiograph views were present and a modified RUST score was able to be determined. Spearman's correlation was used, and a correlation coefficient was accepted as demonstrating at least fair correlation at ≥0.30, moderate correlation at ≥0.60, and very strong correlation at ≥0.80.

## Results

Data from 297 enrolled patients with radiographic follow-up and measured RUST scores were included in the analysis. The most common definitive treatment modality in this group was intramedullary fixation; 226 patients were treated with the SIGN standard nail with 1 or 2 distal interlocking screws, 49 were treated with the SIGN Fin nail (which relies on interference fit for distal fixation), and 14 were treated with the AO universal femoral nail (DePuy Synthes). 5 patients were treated with plate fixation, and 1 was treated with external fixation. In total, 15 patients of the 297 included in this study were recommended to have a revision surgery, for reasons including infection, nonunion, malalignment, and missed interlocking screws.

### Validity and Reliability of Modified Radiographic Union Score for Tibia Score for Femoral Shaft Fractures

**Figure 2:**
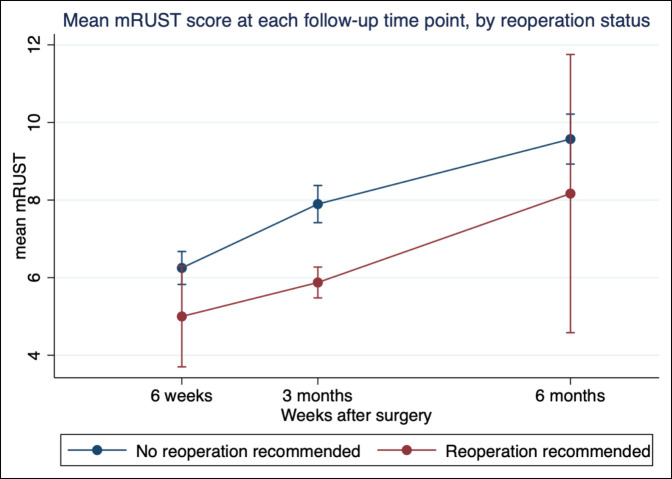
Graph showing the mean mRUST score at each follow-up time point by revision surgery status. mRUST = modified Radiographic Union Score for Tibia.

Fair correlation (defined as SCC ≥0.30) was observed between modified RUST scores and EQ-5D index (SCC 0.417, *P* < 0.001) and EQ-5D VAS scores (SCC 0.343, *P* < 0.001) at all follow-up time points (Table 2). Compared with patients who required revision surgery, patients who did not require revision surgery had significantly higher modified RUST scores at 3 months (mean difference 2.02, *P* < 0.005), and the trend existed at 6 weeks (mean difference 1.25, *P* = 0.187) and 6 months (mean difference 1.40, *P* = 0.323) (Table 3 and Figure 2). Interrater reliability for evaluation of RUST scores between two fellowship trained orthopaedic trauma surgeons (A.N.E., Z.M.W.) was strong (kappa = 0.75, *P* < 0.001) (Table 4).

**Table 2:** Convergent Validity of RUST Scores With Patient-reported Quality of Life Measures

Factor	All Time Points		6 wks		3 mo		6 mo	
	EQ-5D (SCC, *P*, N)	VAS (SCC, *P*, N)	EQ-5D (SCC, *P*, N)	VAS (SCC, *P*, N)	EQ-5D (SCC, *P*, N)	VAS (SCC, *P*, N)	EQ-5D (SCC, *P*, N)	VAS (SCC, *P*, N)
Modified RUST (all 4 cortices)	0.400 (<0.001, N = 222)^a^	0.320 (<0.001, N = 217)^a^	0.330 (0.003, N = 79)^a^	0.119 (0.308, N = 75)	0.299 (0.006, N = 84)^a^	0.256 (0.020, N = 83)^a^	0.370 (0.004, N = 59)^a^	0.286 (0.028, N = 59)^a^
Single-view RUST (anterior + posterior cortices)	0.408 (<0.001, N = 258)^a^	0.334 (<0.001, N = 253)^a^	0.347 (0.001, N = 98)^a^	0.125 (0.232, N = 94)	0.335 (0.001, N = 94)^a^	0.287 (0.005, N = 93)^a^	0.378 (0.002, N = 66)^a^	0.309 (0.012, N = 66)^a^
Single-view RUST (medial + lateral cortices)	0.331 (<0.001, N = 424)^a^	0.316 (<0.001, N = 421)^a^	0.173 (0.024, N = 170)^a^	0.074 (0.345, N = 165)	0.201 (0.013, N = 151)^a^	0.189 (0.020, N = 152)^a^	0.288 (0.003, N = 103)^a^	0.256 (0.009, N = 104)^a^

EQ-5D = EuroQol five dimensions questionnaire, RUST = Radiographic Union Score for Tibia, SCC = Spearman's correlation coefficient, VAS = visual analog scale

a Notable correlation between RUST score and either EQ-5D or VAS score, as determined by SCC.

**Table 3:** Divergent Validity

Factor	6 wks^a^		3 mo^b^		6 mo^a^	
	Mean	Difference	Mean	Difference	Mean	Difference
Modified RUST (all 4 cortices)						
NR	6.25 (N = 76)	1.25 (*P* = 0.187)	7.90 (N = 82)	2.02 (*P* < 0.005)^c^	9.57 (N = 56)	1.40 (*P* = 0.323)
R	5 (N = 4)		5.88 (N = 4)		8.17 (N = 3)	
Single-view RUST (anterior + posterior cortices)						
NR	3.11 (N = 94)	0.81 (*P* = 0.097)	3.88 (N = 91)	1.28 (*P* < 0.005)^c^	4.70 (N = 63)	0.87 (*P* = 0.219)
R	2.3 (N = 5)		2.6 (N = 5)		3.83 (N = 3)	
Single-view RUST (medial + lateral cortices)						
NR	3.09 (N = 168)	0.65 (*P* = 0.057)	3.93 (N = 152)	1.03 (*P* = 0.01)^c^	4.73 (N = 102)	0.60 (*P* = 0.341)
R	2.44 (N = 8)		2.9 (N = 5)		4.13 (N = 4)	

Mean RUST scores in non-revision surgery vs revision surgery at each time point over 6 months.

NR = nonrevision surgery, R = revision surgery, RUST = Radiographic Union Score for Tibia, Difference = difference between the mean score in NR and R groups with significance of two sample *t*-test

a Assuming equal variances.

b Assuming unequal variances as based on Levene's test for equality of variances.

c Significant.

**Table 4:** Interrater Reliability of RUST Scores

Factor	Modified RUST (All 4 Cortices) N = 203	Single-view RUST (Anterior + Posterior Cortices) N = 244	Single-view RUST (Medial + Lateral Cortices) N = 639
Pearson's *R*	0.902 (sig 0.000)	0.861 (sig 0.000)	0.891 (sig 0.000)
Spearman correlation	0.900 (sig 0.000)	0.865 (sig 0.000)	0.880 (sig 0.000)
Kappa	0.753 (SE 0.044, sig 0.000)	0.732 (SE 0.043, sig 0.000)	0.781 (SE 0.028, sig 0.000)

RUST = Radiographic Union Score for Tibia

### Validity and Reliability of Single-view Radiographic Union Score for Tibia Score

The single-view RUST scores exhibited strong correlation with the modified RUST scores (A/P SCC 0.965 *P* < 0.001, M/L SCC 0.952 *P* < 0.001). Fair correlation was observed between single-view RUST scores and EQ-5D index (A/P SCC 0.421 *P* < 0.001, M/L SCC 0.341 *P* < 0.001) and EQ-5D VAS scores (A/P 0.351 *P* < 0.001, M/L 0.316 *P* < 0.001) at all time points (Table 2). Patients who did not require revision surgery had significantly higher single-view RUST scores at 3 months (A/P *P* < 0.005, M/L *P* = 0.01), and the trend existed at 6 weeks (A/P *P* = 0.097, M/L *P* = 0.65) and 6 months (A/P *P* = 0.219, M/L *P* = 0.341) (Table 3). Interrater reliability for evaluation of single-view RUST scores was strong (A/P kappa = 0.732 *P* < 0.001, M/L kappa = 0.781 *P* < 0.001) (Table 4).

## Discussion

We evaluated radiographs from 297 patients with femoral shaft fractures treated operatively in Dar es Salaam, Tanzania. We found that the modified RUST score demonstrated convergent validity with EQ-5D index and VAS scores, divergent validity between patients requiring revision surgery and those who did not, and excellent interrater reliability, which suggests that this is a valid tool for clinical research in this population. Although the data were not sufficient to validate using an abbreviated single-view RUST score, the results do support that this may be a useful alternative in low-resource settings where both views are not available.

Of note, the mRUST and single-view scores at 3 months exhibited the most notable difference between patients who required revision surgery compared with those who did not. Additional research is needed to establish relevant clinical thresholds for bone healing at different time points, but these data suggest that the mRUST score may allow for earlier identification of healing based on radiographic evaluation at 3 months. This is supported by the work of Lack et al who found that cortical bridging at 4 months discriminates between fractures that will undergo late union without intervention and fractures that are destined for nonunion.^15,16^

To the best of our knowledge, this is the first study exploring the interrater reliability of mRUST scores of femoral shaft fractures in Tanzania. However, our findings are supported by previous studies that have assessed the reliability of RUST scores in other settings and study populations. Francisco and Detoyato^17^ found the RUST score to have high interobserver and intraobserver reliability when applied to radiographs of femoral shaft fractures treated at a tertiary hospital in the Philippines and subsequently uploaded to an online SIGN surgical database before evaluation. Litrenta et al^12^ assessed both the RUST and mRUST scores in patients with distal femur fractures treated with plate or nail fixation at a level I trauma center in the US and found that the mRUST scores had a higher intraclass correlation compared with RUST scores.

Regarding the construct validity of the modified RUST score, our study adds to the existing literature examining the correlation between RUST scores and patient-reported outcomes. Çekiç et al^18^ compared RUST scores in tibial shaft fractures treated with intramedullary nailing with the VAS score as well as the Short Form-36 physical function and pain score and the Karlstöm-Olerud physical function scale and found that RUST scores markedly correlated with these patient-reported outcomes. Although additional research is needed to establish the validity of the use of mRUST scores in isolation, it exhibits promise as an independent measure of patient progress along the healing continuum.

There are some limitations to this study. Some radiographs were of poor quality, and radiographs were not available for all patients at all time points. Although the available radiographs suggested a correlation between the mRUST score and both patient-reported outcomes and complications requiring a revision surgery, more complete imaging data may have established a stronger relationship and allowed for the assessment of longitudinal trends. In addition, although the RUST score was developed with the intention to predict nonunion specifically, the outcome of interest that was measured in the original study was revision surgery for any reason, including complications other than nonunion (such as infection). Our results demonstrated a correlation between the mRUST score and revision surgery for any complication, but inferences about its validity as a predictor of nonunion cannot be made.

## Conclusion

Our study adds to the growing use of RUST scores to assess fractures at sites other than the tibia, by validating the mRUST score as an assessment of fracture healing in diaphyseal femoral fractures in comparison with widely used quality of life measures (EQ-5D/VAS) and showing expected divergence in the setting of complications requiring revision surgery. Apart from being a standardized descriptor of healing, the mRUST score may present utility in determining the need for revision surgery because the presence of callus or bridging on any two cortices, or bridging on one cortex, was found to be correlated with not requiring a revision surgery. Our study also establishes that the validity of a single-view RUST score in low-resource settings may be worth exploring in future studies.
